# Comparative Study of PVDF Sheets and Their Sensitivity to Mechanical Vibrations: The Role of Dimensions, Molecular Weight, Stretching and Poling

**DOI:** 10.3390/nano11071637

**Published:** 2021-06-22

**Authors:** Miroslav Mrlík, Josef Osička, Martin Cvek, Markéta Ilčíková, Peter Srnec, Danila Gorgol, Pavel Tofel

**Affiliations:** 1 Centre of Polymer Systems, Tomas Bata University in Zlín, Třída T. Bati 5678, 760 01 Zlín, Czech Republic; osicka@utb.cz (J.O.); cvek@utb.cz (M.C.); srnec@utb.cz (P.S.); gorgol@utb.cz (D.G.); 2 Polymer Institute, Slovak Academy of Sciences, Dubravská cesta 9, 845 45 Bratislava, Slovakia; 3 Department of Physics and Materials Engineering, Faculty of Technology, Tomas Bata University in Zlín, Vavrečkova 275, 760 01 Zlín, Czech Republic; 4 Department of Physics, Faculty of Electrical Engineering and Communication, Brno University of Technology, Technická 10, 616 00 Brno, Czech Republic; tofel@feec.vutbr.cz; 5 Central European Institute of Technology BUT—Brno University of Technology, Purkynova 656/123, 612 00 Brno, Czech Republic

**Keywords:** poly(vinylidene fluoride), crystallinity, physical properties, vibration sensing, d_33_

## Abstract

This paper is focused on the comparative study of the vibration sensing capabilities of poly(vinylidene fluoride) (PVDF) sheets. The main parameters such as molecular weight, initial sample thickness, stretching and poling were systematically applied, and their impact on sensing behavior was examined. The mechanical properties of prepared sheets were investigated via tensile testing on the samples with various initial thicknesses. The transformation of the α-phase to the electro-active β-phase was analyzed using FTIR after applying stretching and poling procedures as crucial post-processing techniques. As a complementary method, the XRD was applied, and it confirmed the crystallinity data resulting from the FTIR analysis. The highest degree of phase transformation was found in the PVDF sheet with a moderate molecular weight (Mw of 275 kDa) after being subjected to the highest axial elongation (500%); in this case, the β-phase content reached approximately 90%. Finally, the vibration sensing capability was systematically determined, and all the mentioned processing/molecular parameters were taken into consideration. The whole range of the elongations (from 50 to 500%) applied on the PVDF sheets with an Mw of 180 and 275 kDa and an initial thickness of 0.5 mm appeared to be sufficient for vibration sensing purposes, showing a d_33_ piezoelectric charge coefficient from 7 pC N^−1^ to 9.9 pC N^−1^. In terms of the d_33_, the PVDF sheets were suitable regardless of their Mw only after applying the elongation of 500%. Among all the investigated samples, those with an initial thickness of 1.0 mm did not seem to be suitable for vibration sensing purposes.

## 1. Introduction

Structural health monitoring systems are very important components having the capability to monitor the in-service conditions of various large-scale systems that are effectively utilized in the industry [1]. The main advantage of these systems is their capability to monitor various properties using a non-destructive approach. Such systems are effectively applied in many industries, e.g., aerospace [2] or civil engineering [3,4]. It must be stated that usual sensors with proper sensing capabilities applied in the above mentioned applications are mainly based on the ceramics with high density, and therefore weight plays a crucial role here. The utilization of alternative systems with proper vibration sensing capability based on low density materials provides a good alternative [5,6,7].

To address these challenges, various approaches have been utilized to develop self-operating sensors exploiting the energy from natural vibrations such as wind, raindrops, river waves, etc. [8], as well as from random vibrations [9]. In this case, piezoelectric [10], thermoelectric [11] and tribolelectric generators [12] can be applied to fulfill the requested limits of the energy consumption and data harvesting capability while allowing communication with a checking unit. Among the systems mentioned above, the piezoelectric generators have mainly been found to be promising. To construct these elements, various ceramics based on Lead zirconate titanate (PZT) have been effectively applied, which are relatively heavy due to the high density of the ceramics [13]. Due to this reason, the implementation of wireless and lightweight systems is highly appreciated since the weight of the conventional PZT-based sensing systems causes the complicated optimization of, e.g., aircraft structures [14].

Therefore, the utilization of the materials with considerably lower density than that of the PZT-based or other ceramics is of high importance. From this point of view, poly(vinylidene fluoride) (PVDF) seems to be a very promising candidate due to its good piezoelectric activity, low density and high flexibility [15]. The piezoelectricity of this material is not intrinsic; however, it can be induced by various modifications such as stretching [16], poling [17] or acombination of both [18]. In addition, there are various processing techniques to transform the initial α-phase to its electro-active β-phase analogue, such as the addition of particles [19,20], electrospinning [21,22] or melt-electrowriting [23]. There were several studies performed, not specifically on the PVDF, but also on the composite systems which independently and in various conditions showed the piezoelectric activity of the films with various molecular weights: low [24], medium [25,26] and high [27]. How the molecular weight influenced the piezoelectric activity if various molecular weights were applied for the electrospun fiber mats was also recently presented [28]. In this respect, until now and according to our knowledge, there is no detailed research dealing with the effects of the molecular weight (Mw) and initial thickness of the sample on the piezoelectric activity of poling, stretching and the combination of these procedures with respect to the treated samples. Moreover, the benefit of this study is the fact that samples were fabricated in the same conditions and thus can provide a critical overview of the effect of the mentioned parameters on the final vibration sensing capability.

Therefore, this study is mainly focused on the preparation of the PVDF samples with various thicknesses via the compression molding technique and their subsequent subjecting to either the stretching or poling procedure. In addition, the combination of both procedures was applied to the PVDF samples that possessed three various initial thicknesses (0.5, 0.8 and 1.0 mm) and different molecular weights (Mw) (180, 275 and 534 kDa). Different stretching conditions of the samples (50, 100, 200, 300 and 500%) and various poling conditions were also applied to establish a comparative study. The stress–strain curves of the samples were performed. Evaluation of the β-phase was performed using calculation of the peak intensities obtained from the FTIR measurement. Similar characterization of the α-phase to electro-active β-phase was performed using XRD investigations. Finally, the effects of the abovementioned parameters on the vibration sensing activity were evaluated on the basis of d_33_ coefficients.

## 2. Materials and Methods

### 2.1. Materials

Poly(vinylidene fluoride) in the form of pellets with various Mw (180, 275 and 534 kDa) was supplied by Sigma-Aldrich (St. Louis, MO, USA) and was used as received. Silver paste 4929N (DuPont, Stevenage, UK) was applied in the case of electrode patterning for poling and vibration sensing activity measurements. Silicone oil Lukosiol M 50 was used for poling (Ekolube, Brno, Czech Republic).

### 2.2. Fabrication of the Samples and Tensile Texting

The PVDF sheets were produced by the compression molding technique. The desired amounts of the PVDF pellets with the Mw stated above were placed into the metal molds (12 × 12 cm^2^), and the sheets of three different thicknesses (0.5, 0.8 and 1.0 mm) were fabricated. In particular, the mold loaded with the pellets was pre-heated for 10 min until the pellets melted down completely. Then, the mold was closed to achieve the final thickness of the samples and compressed for an additional 5 min. Subsequently, the mold assembly was transferred into a cold press and cooled down to the laboratory temperature in a controlled manner. The materials subjected to the investigation and temperatures of pressing are summarized in Table 1. 

**Scheme 1:**
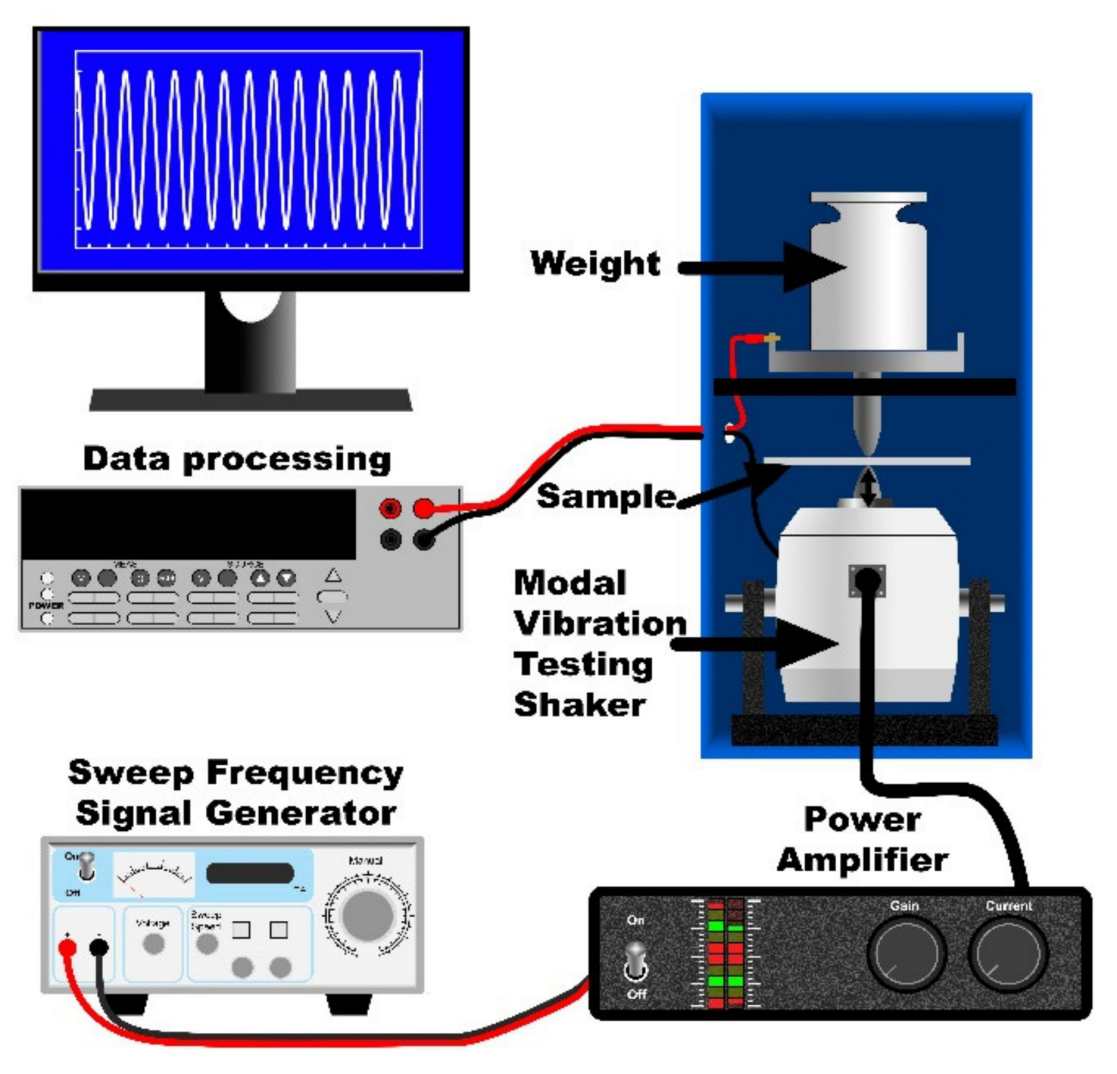
Schematic illustration of the measuring setup for d33 piezoelectric charge coefficients.

**Figure 1:**
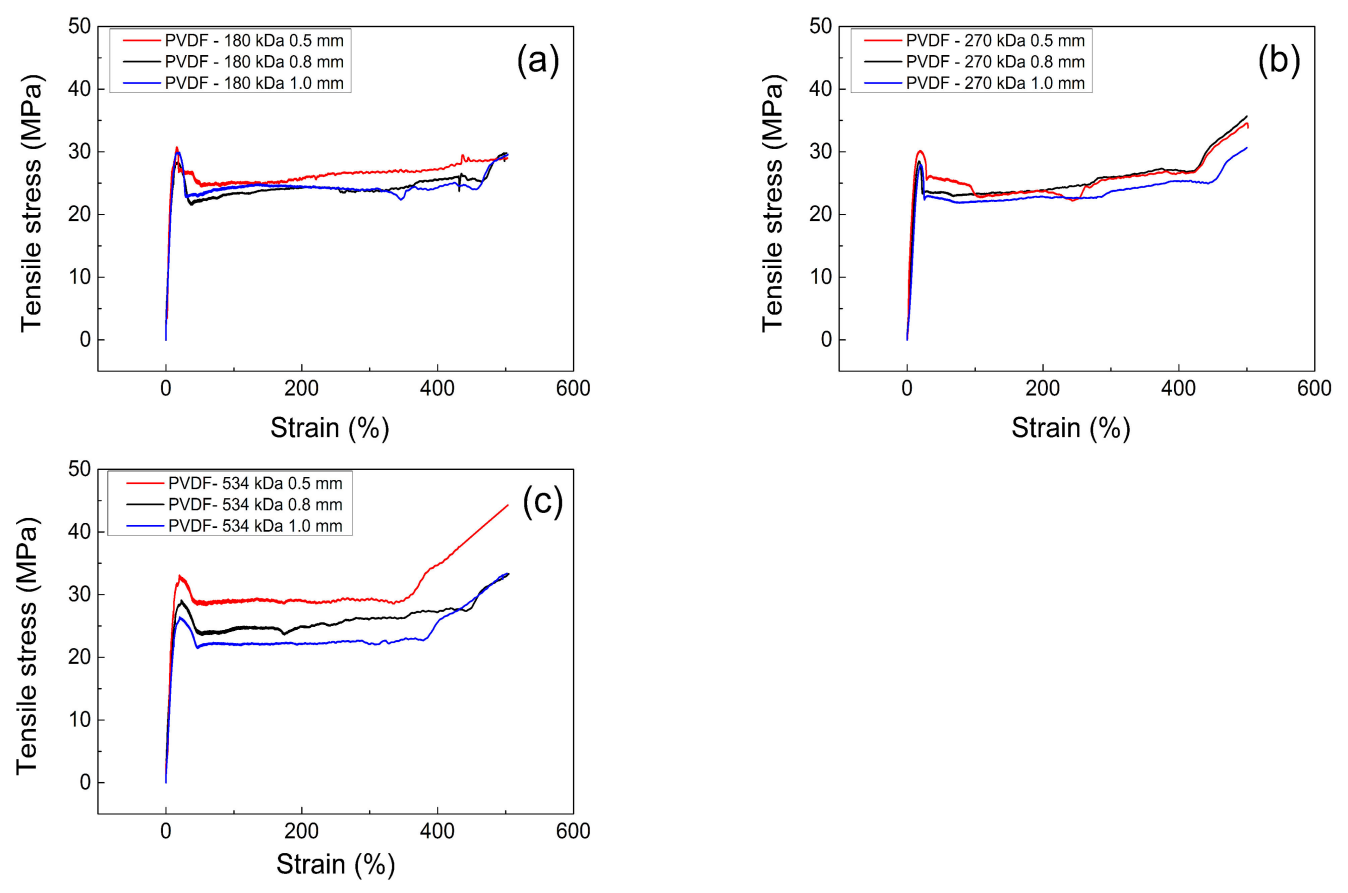
Dependence of the tensile stress on the strain for the PVDF samples with various Mw and the initial thickness of 0.5 mm (red), 0.8 mm (black) and 1.0 mm (blue) (**a**) 180 kDa (**b**) 270 kDa (**c**) 534 kDa.

Then, the PVDF sheets were cut into rectangular strips (100 × 20 mm^2^) by a precise cutting knife and into circles of 30 mm in a diameter by a CO_2_ laser BRM-6090-100 (BRM Lasers, Winterswijk, The Netherlands). Each strip was attached between two clamps (clamp distance of 50 mm) in a climate chamber (Omron, Kyoto, Japan) coupled with the tensile-testing machine MT350-5CT (Testometric, Rochdale, UK). The chamber was heated to 60 °C, and each strip was kept there for 10 min to equilibrate the temperature. Finally, there were two batches of PVDF samples; the first was stretched from 0% up to 500% in one test (these are shown in Figure 1) to see the structural changes over the elongation, and the second batch of the samples were stretched stepwise to different relative elongations of 50, 100, 200, 300 and 500%, and subsequently poled according to the procedure below. In all measurements, the cross-head speed was 10 mm·s^−1^. For each measurement (Scheme 1) and each deformation, at least 10 samples were prepared.

### 2.3. Polarization of the Samples

The conductive silver paste was spread from both sides onto the elongated PVDF strips and dried for 24 h at room temperature (RT). Prepared materials were placed between two electrodes that were immersed into the pre-heated silicone oil bath (110 °C). The electric field of 7 kV·mm^−1^ was applied for 30 min. In this procedure, high voltage power supply PS365 (Stanford Research Systems, Sunnyvale, CA, USA) was used as the electric field source. Afterwards, the samples were extracted from the oil bath and cooled down to RT by an electric drying device; the electric field was finally switched off. On the polarized samples, the d_33_ coefficient was measured ten times, and the average values are presented below.

### 2.4. Characterization Methods

The phase transformation from the α- to β-phase was investigated on the prepared samples with various molecular weights, initial thicknesses and the elongations using the Fourier-Transform Infrared (FTIR) spectroscopy on the Nicolet 6700 (Thermo Scientific, Waltham, MA, USA) device after employing the ATR accessory with the Germanium crystal. The data were collected in the wavenumber range from 4000 to 500 cm^−1^. Moreover, the β-phase content, F(β), was calculated according to the following Equation (1) as also shown elsewhere [20,23]:

(1) $$F(\beta )\;=\;\frac{A_{\beta }}{\left(\frac{K_{\beta }}{K_{\alpha }}A_{\alpha }{+A}_{\beta }\right)}$$

where *A*_α_ and *A*_β_ are the absorbance values corresponding to the wavenumber of 762 cm^−1^ and 840 cm^−1^, respectively. The *κ*_α_ and *κ*_β_ are the absorption coefficients for the α-crystalline phase and β-crystalline phase, having values of 6.1 × 10^4^ cm^2^ mol^−1^ and 7.7 × 10^4^ cm^2^ mol^−1^, respectively.

The crystalline phase development, in terms of the α-phase and β-phase, was investigated in the prepared sheets using the X-Ray diffractometer (XRD) MiniFlex600 (Rigaku, Tokyo, Japan) with Co-Kα radiation source (operating at 40 kV and 20 mA) and a scan range 2θ between 5° and 45°. Due to the employment of the Co-Kα source with different working characteristics (λ = 1.789 Å) from the conventional Cu-Kα source (λ = 1.541 Å), the angle 2θ for the α-phase and β-phases was slightly shifted towards higher values.

Vibration sensing capabilities were investigated by measuring the charge piezoelectric coefficient d_33_ on the electrometer 6517b (Keithley, Solon, OH, USA) as has been similarly published in preceding studies [29,30,31,32]. The mechanical force oscillations were induced by a vibration test system TV 50018 (Tira, Schalkau, Germany) and detected by a sensor 208C01 (PCB Piezotronics, Hückelhoven, Germany). The experimental setup is schematically shown in Scheme 1. In all cases, the PVDF samples were metalized by the silver paste from both sides and were clamped between two measuring probes with static force 0.5 N; the upper probe was clamped by a bolt, and its position was static during measurement, while the bottom probe was excited by a harmonic mechanical oscillation force of peak to peak value 0.25 N with a frequency of 110 Hz. This harmonic oscillation force was applied onto the PVDF and the electric charge generated by the sample was recorded in a time domain containing ten periods of the measured signal with the same frequency as the mechanical excitation 110Hz. Average minimal and maximal values were then evaluated from the recorded signal, and their different values represented an electric charge generated by the sample. A static offset was not taken into account in calculations. Only the harmonic signal and its peak-to-peak values were taken into account. Parasitic noise was significantly reduced by using the shaker and the measured sample in an electrically shielded box. The piezoelectric charge coefficient d_33_ (pC N^−1^) was evaluated using Equation (2), which can be read as follows:

(2) $$d_{33}=\frac{Q}{F}$$

where *Q* is the electric charge generated by the sample, and *F* is the excitation force applied on the sample. Using this technique, each sample was analyzed ten times in slightly different positions of its metalized area, and the resulting piezoelectric charge coefficient d_33_ was represented as the average value of these obtained values.

## 3. Results and Discussion

The tensile properties of the PVDF strips were investigated using the tensile testing machine. As can be seen from Figure 1, all samples were able to sustain the relative elongations up to 500%, as was similarly observed by other researchers performing 500% elongation on the PVDF films with Mw 125 kDa [33]. As expected, the Mw played a crucial role in mechanical properties since the tensile stress increased with the increasing Mw. All samples exhibited a yield point; moreover, the additional stretching above 400% led to changes of the stress–strain curve slope, showing that the additional stress-induced crystallization manifested inside the samples. The PVDF with the Mw of 534 kDa exhibited, after the re-orientation, the highest values of the tensile stress at break. Interestingly, the samples with the initial thickness of 0.5 mm showed the best mechanical performances, mainly due to more intensified orientation which was significantly influenced by the lowest initial thickness. The shape of the curves supports the hypothesis, as further discussed in this paper, that the initial thickness is also a very important parameter remarkably affecting the final vibration sensing capability of the PVDF sensors.

**Figure 2:**
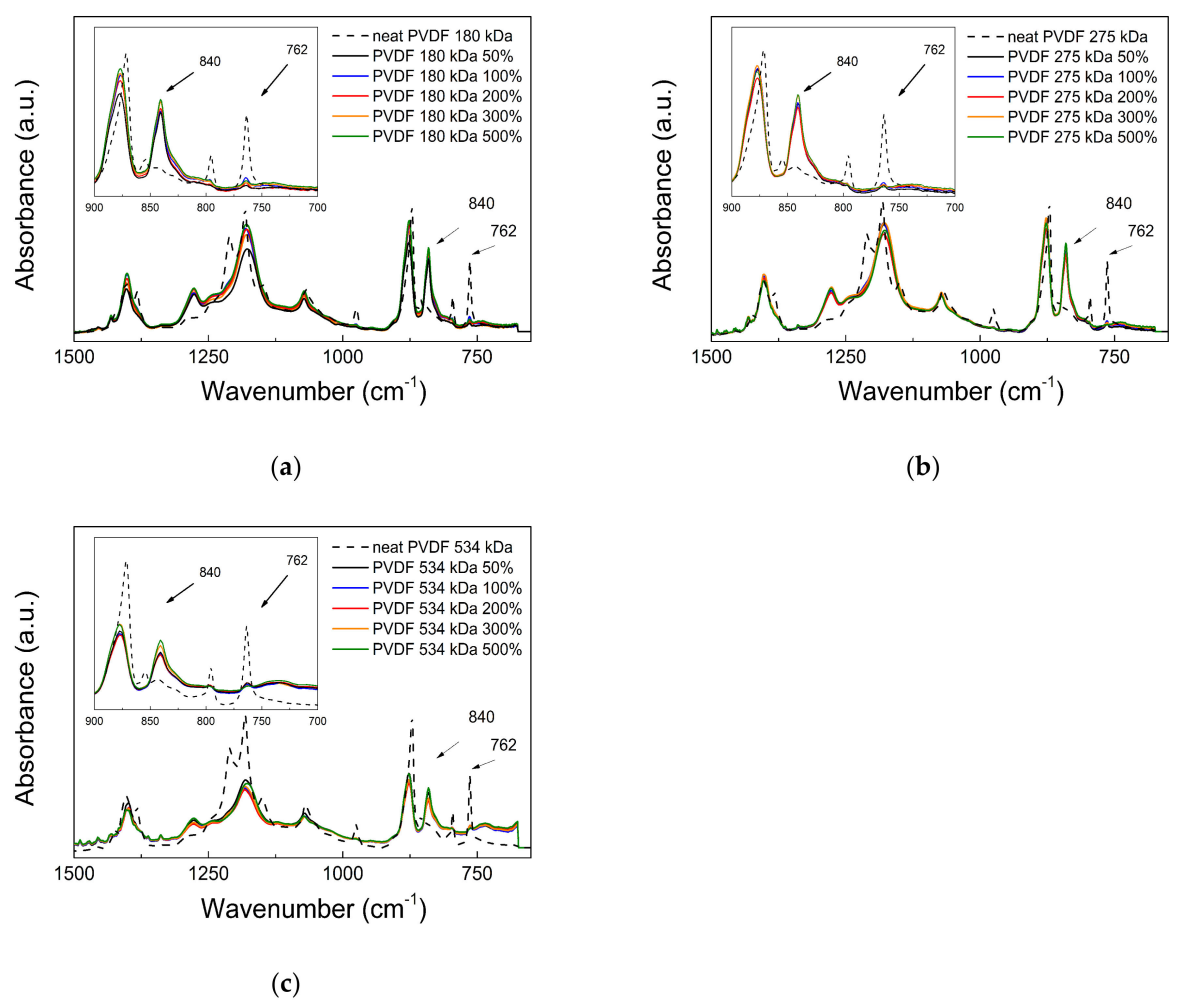
FTIR spectra of the selected samples with initial thickness of 0.5 mm and various molecular weights (**a**)180 kDa (**b**) 270 kDa (**c**) 534 kDa.

In order to investigate the transformation of the non-electroactive α-phase to its electroactive β-phase analogue, the FTIR spectra were collected and investigated as carried out elsewhere [34,35,36]. Non-treated PVDF sheets showed in all cases the co-existing presence of both the α-phase and β-phase. Interestingly, both the poling and stretching treatments, when applied individually, induced the transformation of the α-phase into the β-phase. Similar behavior was observed when the combination of both treatments was utilized; however, as was revealed later, this procedure was not reflected in the vibration sensing properties, which means that stretching and poling procedures must be applied in tandem to induce the piezoelectric response of the samples. Figure 2a–c was plotted to interpret the implications of the thickness of the individual PVDF sheets with various Mw. As can be seen (Table 2), significant phase transformation was distinguishable after subjecting the samples to the relative elongation of 50%. However, significantly more developed structures of the β-phase were visible after applying the relative elongation of 300% and higher. For the sake of better understanding, only the data related to the samples with the best vibration sensing capabilities are displayed. In order to evaluate the effect of the initial thickness of the phase transformation process, Equation (1) was used to calculate the corresponding β-phase content. Consequently, the highest F(β) values were obtained for the samples with the lowest initial thickness, and they gradually increased in line with the tensile deformation. For all samples subjected to the relative elongation of 500%, the β-phase content was the highest, exhibiting 88.3, 90.8 and 90.4 for the PVDF samples having an Mw of 180, 275 and 534 kDa, respectively. Such results are in good agreement with well developed β-phase presented in other studies [32,37,38]. At this point, it should be emphasized that the lower sample thickness acted similarly due to the presence of the embedded particles; the existence of the steric obstacle intensified the deformation and thus stress-induced re-organization of the crystalline phase, which contributed to the enhanced β-phase development [39]. This finding corresponds well with the results from the tensile testing (Figure 1) and with vibration sensing data shown below. To discuss the obtained data, the study dealing with PVDF samples of various Mws [28] is in fact the same as that reported in this study in which the authors fabricated the samples using electrospinning. It can be seen that in electrospinning, Mw plays a more crucial role in β-phase development since the difference between the 180 kDa and 530 kDa was 8.3%, while in our case it was just 2.1%. Finally, the results for β-phase content are very similar to those obtained for low Mw (125 kDa) samples elongated to 500% [33].

**Figure 3:**
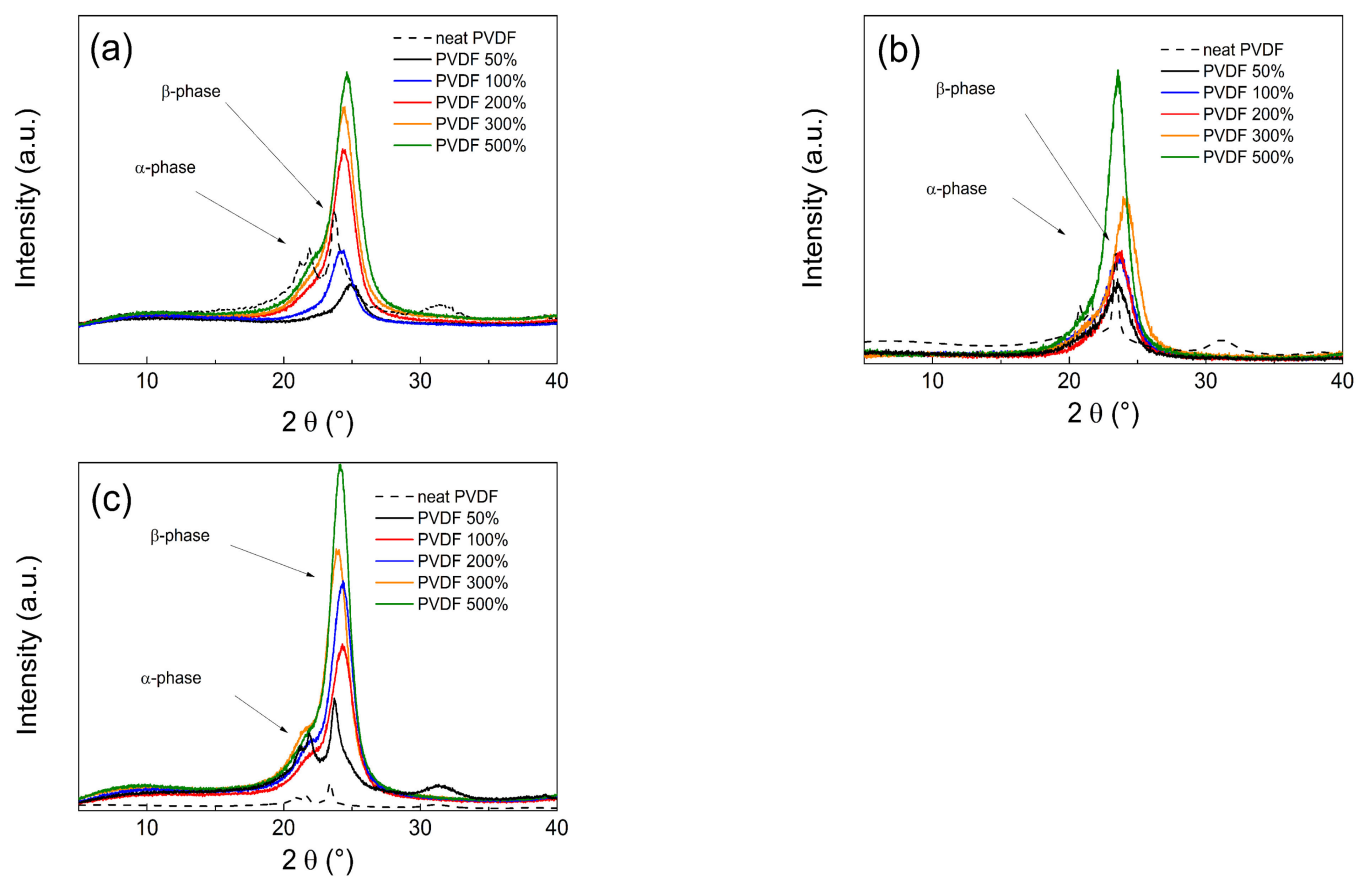
XRD spectra of the PVDF samples with the initial thickness of 0.5 mm and Mw of 180 kDa (**a**), 275 kDa (**b**) and 534 kDa (**c**).

In order to confirm the data obtained from the FTIR analysis, the XRD patterns were collected for all the investigated PVDF samples. For a clearer presentation of the results, we present the data acquired from the samples with the initial thickness 0.5 mm (Figure 3a–c). As can be seen, the non-treated PVDF sheets unambiguously showed a significantly developed α-phase with two typical diffraction lines in the 2θ region from 20.7° to 21.8°, and a β-phase ranging from 23.4° to 23.6° depending on the Mw. In correlation with the mechanical analysis, the individual poling and stretching procedures led to the transformation of the crystalline structure, but with no impact on the vibration sensing capability. In this case, the mutual application of these two processes yielded samples with a similar structure to those mentioned, but with direct impact on the sensing capability. Therefore, the α-phase was significantly suppressed by the treating process, and the position of the corresponding peaks was visible only as a shoulder of the main peak that was even dismissed after applying the subsequent elongation. There was just one exception in this trend that occurred for the PVDF with an Mw of 534 kDa when elongated by 50%. In this specific case, the α-phase was still persistent, mainly due to the fact that high-Mw PVDF requires more energy (deformation) for a better development of the β-phase since long polymer chains are tougher as was shown in the tensile testing. For the rest of the samples, the β-phase peak was visible in the 2θ range from 23.6° to 24.6° where it was randomly shifting depending on the executed elongation. The height of the peak indicated a higher portion of the β-phase that increased with the elongation and achieved the highest intensity for the relative elongations of 500%. Such results correspond well with the previous investigations and will be further correlated with the vibration sensing capabilities.

The systematic characterization of the PVDF sheets performed in this paper gradually contributes to the hypothesis that Mw, initial thickness and stretching conditions are relevant factors that significantly influence structural properties and thus final vibration sensing activity. From this point of view, all samples were investigated using the measuring setup depicted in Scheme 1.

**Figure 4:**
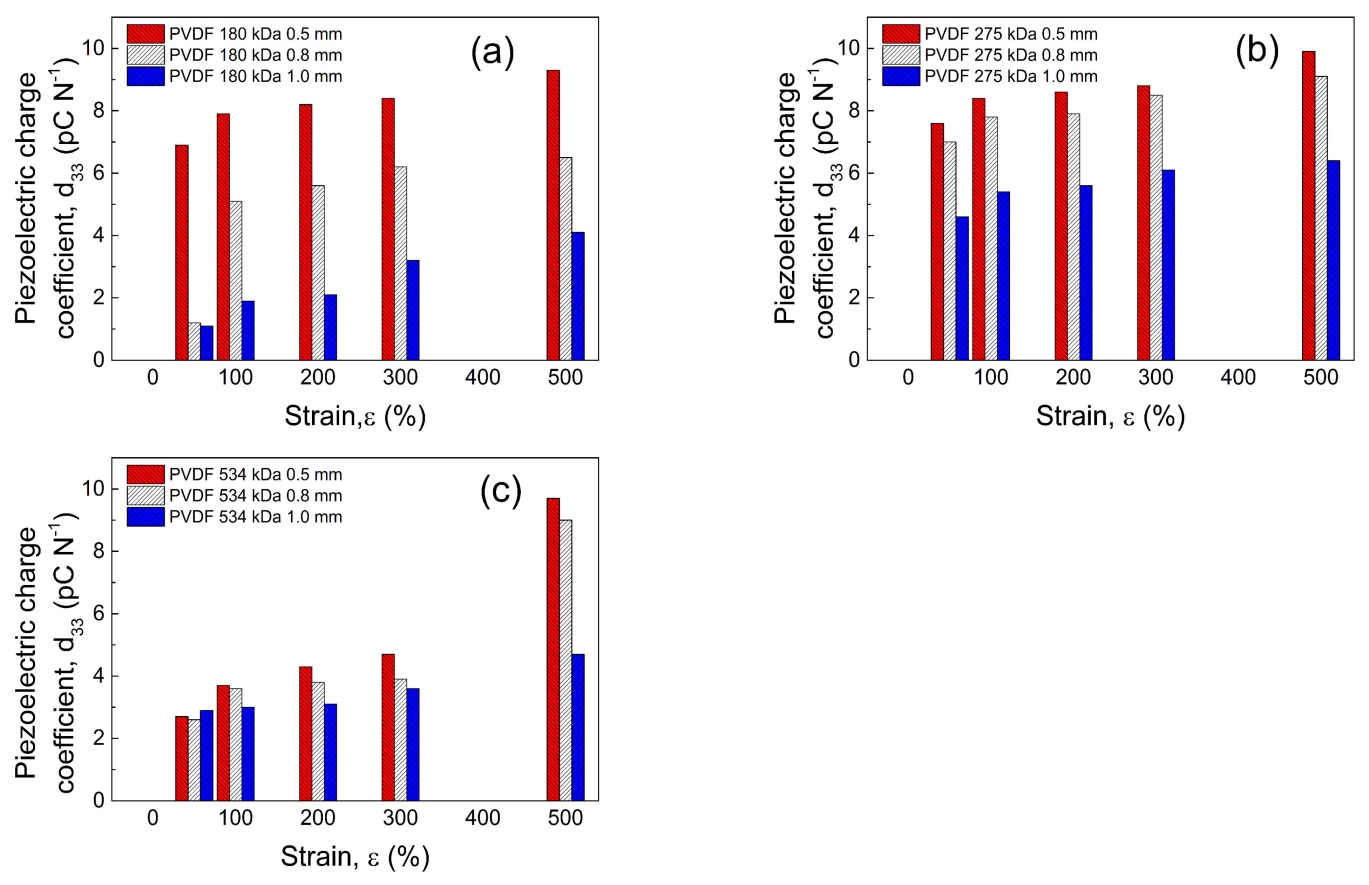
d_33_ piezoelectric constant identification at various strain deformations for PVDF samples of different molecular weights and initial thicknesses. 180 kDa (**a**), 275 kDa (**b**) and 534 kDa (**c**).

It was found that the PVDF samples individually poled or stretched were not able to collect any signal or provided very noisy signals for the calculation of the actual d_33_ piezoelectric charge coefficients and were not included in Figure 4. All performed experiments with negligible or no piezo activity are summarized in Tables S1 and S2 as part of the Supporting Information (SI). On the contrary, the actual d_33_ values, some of them even lower than 4 pC N^−1^, were able to be calculated for the elongated samples and are summarized in the table. From the precisely calculated d_33_ values, it can be concluded that Mw appears to be a highly relevant parameter affecting the vibration sensing behavior of the PVDF. The d_33_ coefficients of the PVDF samples appeared suitable for the vibration sensing regardless of their Mw; however, the PVDF of the highest Mw could be applicable only after subjecting to the elongation of 500%. In the case of the PVDF samples with an Mw of 180 and 275 kDa, the suitable d_33_ values were generally achieved from relative elongations above 50%. At this point, it is, however, necessary to include another important factor, which is represented by the initial thickness. For the PVDF with an Mw of 534 kDa, only thicknesses of 0.5 mm and 0.8 mm elongated by 500% provided sufficient d_33_ values due to high steric hindrance and higher energy requirements for the phase transformation process. On the other hand, the sample thickness of 1.0 mm appeared to be unsuitable for the samples with an Mw of 180 and 275 kDa. The samples composed of the PVDF with an Mw of 275 kDa exhibited the best performance and thus a reasonable vibration sensing capability since the d_33_ values obtained for the thicknesses of 0.5 mm and 0.8 mm were valid from 50% deformation and the attained values of 7.6 pC N^−1^ and 7.0 pC N^−1^, respectively, while at 500% deformation they reached values of 9.9 pC N^−1^ and 9.1 pC N^−1^, respectively. 

In order to critically discuss our findings, the results from other published papers dealing with PVDF and its piezoelectric activity were obtained. In this respect, the neat PVDF film prepared by solvent casting with an initial thickness of 120 μm provided 7 pC/N [40], which is very similar to those with 500 μm initial thickness. Where an even thickness (less than 100 μm) of samples is provided, i.e., utilizing electrospun fiber mats of neat PVDF showing 22.6 pC/N with 84% of electro-active β-phase [41], it can be easily estimated that the lower the thickness, the greater the piezoelectric charge coefficient. However, the utilization of the various techniques such as solvent casting, electrospinning and compression molding does not clearly prove this statement. Therefore, it is our intention to conduct a similar study to that performed in this paper, but using the extrusion sheet molding which provides a variety of initial thicknesses from 0.1 mm to 1 mm, while compression molding has certain limitations in thickness precision, especially at a thickness of less than 500 μm.

## 4. Conclusions

This paper clearly shows that various parameters such as Mw, initial sample thickness and stretching ratio significantly affected the vibration sensing capability of the PVDF sheets. In this respect, the tensile elongation tests showed that stress-induced crystallization took place in all of the investigated samples, and the best mechanical properties were found for the PVDF having an Mw of 534 kDa. Other important factors determining the transformation of the α-phase into the electroactive β-phase included the initial thickness of the PVDF sheets and their poling and stretching. In this respect, the best properties were found for those samples with the lowest thickness of 0.5 mm and the highest elongation of 500%; all attained approximately 90% of the β-phase content. These results correlate with the XRD data showing that the peaks of the crystalline β-phase reached a much higher intensity as well as corresponding peak areas. In order to determine the impact of the molecular/processing parameters on the vibration sensing capability, the d_33_ piezoelectric charge coefficients were calculated. It was clearly found that procedures (either poling or stretching) do not work individually and have to be combined, otherwise the signals from the excitation vibrations are below the detection limit or they are too noisy. It was also confirmed that the lower the initial sample thickness, the higher the vibration sensing capability obtained. On the other hand, the higher the deformation performed, the higher the vibration sensing activity received. Therefore, the critical parameters, initial thickness with Mw and stretching, provided the best results; specifically, the PVDF sample with an initial thickness of 0.5 mm and an Mw of 275 kDa exhibited, after subjecting to relative elongation of 500%, the highest d_33_ reaching a value of 9.9 pC N^−1^ under the presented conditions during this research. Finally, it can be concluded that the performed investigation provides a comparative evaluation of various effects on the vibration sensing capability and shows that all these critical parameters have to be taken into serious consideration when designing a sensing system based on PVDF.

## Figures and Tables

**Table 1:** Summarized labeling of the prepared samples.

Sample Code	Average Mw (kDa)	Temperature and Thickness of the Samples		
		0.5 mm	0.8 mm	1.0 mm
PVDF 180	180	190 °C	190 °C	190 °C
PVDF 275	275	190 °C	190 °C	190 °C
PVDF 534	534	205 °C	205 °C	205 °C

**Table 2:** Summarized values of the β-phase content (F(β)) calculated according to Equation (1).

	Applied Strain (%)					
**Sample code**	0	50	100	200	300	500
PVDF 180 kDa 0.5 mm	21.5	76.9	83.1	85.8	86.4	88.3
PVDF 180 kDa 0.8 mm	21.2	28.6	73.4	76.2	78.7	81.5
PVDF 180 kDa 1.0 mm	21.8	27.4	29.5	31.1	32.9	69.4
PVDF 275 kDa 0.5 mm	22.3	81.7	85.4	86.3	88.2	90.8
PVDF 275 kDa 0.8 mm	22.4	78.6	82.1	84.5	86.2	87.1
PVDF 275 kDa 1.0 mm	22.7	59.6	65.3	66.2	71.4	72.6
PVDF 534 kDa 0.5 mm	24.7	33.1	52.7	56.3	63.1	90.4
PVDF 534 kDa 0.8 mm	25.1	37.2	50.4	51.2	61.2	87.9
PVDF 534 kDa 1.0 mm	24.3	31.9	49.2	50.8	61.8	71.2

## Supplementary Materials

The following are available online at https://www.mdpi.com/article/10.3390/nano11071637/s1, Table S1: Summarized values of piezoelectric charge coefficient d33 for 0% deformation at various applied voltages, Table S2: Summarized values of piezoelectric charge coefficient d33 for 0 kV/mm at various applied deformations.
