# Supplementary material for: Comparative Study of PVDF Sheets and Their Sensitivity to Mechanical Vibrations: The Role of Dimensions, Molecular Weight, Stretching and Poling
Source: Nanomaterials (Basel). 2021 Jun 22;11(7):1637. doi: 10.3390/nano11071637 (PMC8305835; doi:10.3390/nano11071637)
Supplement: Supplementary file 1 [file nanomaterials-11-01637-s001.zip › nanomaterials-1235719-supplementary.pdf]

nanomaterials

MDPI

Supporting Information

Comparative Study of PVDF Sheets and Their Sensitivity to Mechanical Vibrations: The Role of Dimensions, Molecular Weight, Stretching and Poling

Miroslav Mrlík [1,*], Josef Osička [1], Martin Cvek [1], Markéta Ilčíková [1,2,3,*], Peter Srnec [1], Danila Gorgol [1], Pavel Tofel [4,5]

[1] Centre of Polymer Systems, Tomas Bata University in Zlín, Třída T. Bati 5678, 760 01 Zlín, Czech Republic; osicka@utb.cz (J.O.); cvek@utb.cz (M.C.); srnec@utb.cz (P.S.); gorgol@utb.cz (D.G.)
[2] Polymer Institute, Slovak Academy of Sciences, Dubravská cesta 9, 845 45, Bratislava 45, Slovakia
[3] Department of Physics and Materials Engineering, Faculty of Technology, Tomas Bata University in Zlín, Vavrečkova 275, 760 01 Zlín, Czech Republic
[4] Brno University of Technology, Department of Physics, Faculty of Electrical Engineering and Communication, Technická 10, 616 00, Brno, Czech Republic; tofel@feec.vutbr.cz
[5] CEITEC BUT – Brno University of Technology, Purkynova 656/123, 612 00 Brno, Czech Republic
* Correspondence: mrlik@utb.cz (M.M.); ilcikova@utb.cz (M.I.)

Table S1. Sumarized values of piezoelectric charge coefficient d_{33} for 0% at various applied voltages.

	Applied voltage (kV mm^{-1})					
**Sample code**	0	0.5	1	3	5	7
PVDF 180 kDa 0.5 mm	N/A	N/A	N/A	0.1	0.2	0.4
PVDF 180 kDa 0.8 mm	N/A	N/A	N/A	N/A	N/A	N/A
PVDF 180 kDa 1.0 mm	N/A	N/A	N/A	N/A	N/A	N/A
PVDF 275 kDa 0.5 mm	N/A	N/A	0.1	0.2	0.4	0.4
PVDF 275 kDa 0.8 mm	N/A	N/A	N/A	N/A	N/A	N/A
PVDF 275 kDa 1.0 mm	N/A	N/A	N/A	N/A	N/A	N/A
PVDF 534 kDa 0.5 mm	N/A	N/A	N/A	0.1	0.1	0.3
PVDF 534 kDa 0.8 mm	N/A	N/A	N/A	N/A	N/A	N/A
PVDF 534 kDa 1.0 mm	N/A	N/A	N/A	N/A	N/A	N/A

*N/A denotes the piezoelectric charge coefficient d_{33} values under the detection limit.

Table S2. Sumarized values of piezoelectric charge coefficient, d_{33} for 0 kV mm^{-1} at various applied deformations.

	Applied strain (%)					
**Sample code**	0	50	100	200	300	500
PVDF 180 kDa 0.5 mm	N/A	N/A	N/A	N/A	0.1	0.1
PVDF 180 kDa 0.8 mm	N/A	N/A	N/A	N/A	N/A	N/A
PVDF 180 kDa 1.0 mm	N/A	N/A	N/A	N/A	N/A	N/A
PVDF 275 kDa 0.5 mm	N/A	N/A	N/A	N/A	0.1	0.3
PVDF 275 kDa 0.8 mm	N/A	N/A	N/A	N/A	N/A	N/A
PVDF 275 kDa 1.0 mm	N/A	N/A	N/A	N/A	N/A	N/A
PVDF 534 kDa 0.5 mm	N/A	N/A	N/A	N/A	0.1	0.2
PVDF 534 kDa 0.8 mm	N/A	N/A	N/A	N/A	N/A	N/A
PVDF 534 kDa 1.0 mm	N/A	N/A	N/A	N/A	N/A	N/A

*N/A denotes the piezoelectric charge coefficient d_{33} values under the detection limit.
